# Concepts of social epidemiology in health services research

**DOI:** 10.1186/s12913-015-1020-z

**Published:** 2015-09-02

**Authors:** Olaf von dem Knesebeck

**Affiliations:** Department of Medical Sociology, University Medical Center Hamburg-Eppendorf, Martinistr. 52, D-20246 Hamburg, Germany

## Abstract

**Background:**

Social epidemiologists aim to identify social characteristics that affect the pattern of disease and health distribution in a society and to understand its mechanisms. Some important concepts of social epidemiology are: social inequalities, social relationships, social capital, and work stress.

**Discussion:**

Concepts used in social epidemiology can make a useful contribution to health services research because the underlying social factors do not only influence health but are also related to health care. Social inequality indicators like education or income have an impact on access to health care as well as on utilization and quality of health care. Social relationships influence adherence to medical treatment, help-seeking behavior, utilization of health services, and outcomes. Social capital in health care organizations is an important factor for the delivery of high-quality coordinated care. Job stress is highly prevalent among health care providers and can not only affect their health but also their performance.

**Summary:**

The theoretical considerations behind factors like social inequalities, social relationships, social capital and work stress can enrich health services research because theory helps to specify the research question, to clarify methodological issues, to understand how social factors are related to health care, and to develop and implement interventions.

## Background

A systematic analysis of social factors is crucial for health services research as these factors affect access to health care, the quality of health care, and ultimately health and well-being [1]. Social sciences have a long tradition in dealing with origins, manifestation, impact and changes of social factors while social epidemiology studies the social distribution and social determinants of states of health. Social epidemiology proposes to identify social characteristics that affect the pattern of disease and health distribution in a society and to understand its mechanisms. Some important concepts of social epidemiology are: social inequalities, social relationships, social capital, and work stress [2, 3]. These factors are inter-related and have been repeatedly found to be significantly associated with different health outcomes.

Health services research has been criticized for often using atheoretical approaches that tend to result in a simple input/output, or black box type of study. Moreover, it has been stated that a stronger theoretical base would help health services research to be more informative and influential [4]. The main argument of this paper is that concepts of social epidemiology can make a useful contribution to health services research because the underlying social factors do not only influence health but also have an impact on access to health care as well as on utilization and quality of health care. Therefore, the theoretical considerations behind factors like social inequalities, social relationships, social capital or work stress can also provide a theoretical framework for studies in health services research.

In the following, four concepts of social epidemiology are introduced by outlining their theoretical background and impact on health. Thereafter, the potential relevance for health services research is delineated and discussed.

## Discussion

### Social factors: theoretical considerations and impact on health

#### Social inequality

Social inequality can be defined as the unequal distribution of goods, services and opportunities within a group or society. Based on the concept of social class by Max Weber [5], social epidemiologists frequently use indicators of ‘life chances’ such as education, occupation and income to measure social inequality. “The assumption here is that it is mechanisms linked to aspects of distribution that are most important for health – the skills, knowledge, and resources held by individuals that form the key linkage between social stratification and the health of those individuals” [6]. Therefore, studies in social epidemiology dealing with health inequalities foremost focus on the association between these indicators of socioeconomic position (education, occupation and income) and health (e.g. [7]). Generally, these studies have consistently shown a social gradient of health, i.e. the lower an individual’s socioeconomic position the worse their health [8].

Such social inequalities in health should be distinguished from biological differences. If health differences are attributable to biological variations or free choice it may be impossible or unacceptable to change the health determinants and so the health inequalities are unavoidable. If instead health inequalities are attributable to the external environment and conditions mainly outside the control of the individual, these inequalities are considered avoidable and unfair and lead to inequity in health. According to the capabilities approach of Amartya Sen, it is the lack of opportunity to achieve good health because of inadequate social arrangements that is crucial for health inequity [9].

#### Social relationships

Following the work of Émile Durkheim [10] on social integration, alienation and anomie, the health effects of social relationships have been systematically studied since the 1970s [11]. There are different approaches to define and measure social relationships covering three major components [12]: (a) the degree of integration in social networks, (b) the social interactions that are intended to be supportive (i.e., received social support), and (c) the beliefs and perceptions of support availability held by the individual (i.e., perceived social support). In this regard, social networks represent the structural aspects of social relationships and social support represents the functional aspects. Moreover, social support is typically divided into subtypes, which include emotional (e.g. understanding, esteem) and instrumental support (e.g. practical help, financial support). There is considerable evidence that social integration and social support are beneficial to health. For example, a meta-analysis found that the influence of structural and functional aspects of social relationships on risk for mortality is comparable with well-established risk factors [12]. Overall, a 50 % increased likelihood of survival as a function of stronger social relations was found. Odds ratios were 1.9 for social integration, 1.5 for social networks, 1.4 for perceived social support and 1.2 for received social support.

#### Social capital

Based on the works of Pierre Bourdieu [13], James Coleman [14] and Robert Putnam [15] the concept of social capital was first used in social epidemiology in 1997 to analyze regional differences in mortality in the US [16]. There is no consensual definition of the concept and, especially in the approach of Bourdieu, there is a conceptual overlap with social relationships. Broadly speaking, social capital can be defined as those features of social structures – such as levels of interpersonal trust and norms of reciprocity, civic engagement, and mutual aid - which act as resources for individuals and facilitate collective action [17]. According to Macinko and Starfield [18] social capital may function on different levels: the macro-level (i.e. historical, social, political, and economic features of societies), the meso-level (i.e. features of organizations and neighborhoods), individual behaviors (e.g. social participation), and individual norms (e.g. trust and reciprocity). Positive associations between social capital and health were repeatedly found on aggregate level (i.e. in ecological studies) and on individual level as well as in multilevel studies [19, 20]. However, depending on the concept and measure of social capital used, studies on health effects show some inconsistencies.

#### Work stress

Work and employment are of critical importance for health. The demand-control model and the effort-reward imbalance (ERI) model are two influential theoretical approaches that aim to identify stressful working conditions which are likely to adversely affect the health of working people. The demand-control model postulates that job strain results from the combination of high (quantitative) job demands and low job control that is subdivided into skill discretion and decision authority [21]. The ERI-model is focused on the experienced lack of social reciprocity [22]. An imbalance between high efforts and low rewards in terms of esteem, salary, job promotion or job security leads to negative emotions and harmful stress. Both models assume that job stress leads to reduced health. This assumption was confirmed in numerous studies [23–25].

According to the theory of fundamental causes of disease [26], social inequality is considered as the underlying fundamental social condition determining health and, resources as well as risk factors such as social relationships, social capital and psychosocial work stress can be regarded as intermediate social factors in the pathways between social inequality and health outcomes [27].

### Relevance for health services research

#### Social inequality

The concept of social inequality is important for health services research because it was shown that social inequality indicators like education or income have an impact on *access* to health care as well as on *utilization* and *quality* of health care in different countries/health care systems [28]. In terms of inequality in access, it is useful to differentiate between horizontal and vertical equity [29]. Horizontal equity means that persons with equal needs have equal access to care, whereas vertical equity means that those with unequal needs (e.g. people with a low socioeconomic position and increased morbidity risks have more needs) have appropriately unequal (advanced) opportunities to access health care. However, contrary to vertical equity, the inverse care law proposed by Tudor Hart says that the availability of good medical care tends to vary inversely with the need for it in the population served [30]. In many studies on health care inequalities, utilization is used as a proxy for access. However, while access is a characteristic of the supply side, utilization is more a patient attribute. Inequality in utilization may be due to varying preferences or due to differing opportunities of patients (e.g. information about adequate health services). The former are often interpreted as acceptable while the latter are considered unacceptable/inequitable [29]. Studies exploring socioeconomic differences in the quality of health care often focus on inequalities in health outcomes like mortality or quality of life. However, interpretation of respective findings is difficult because it is unclear whether observed differences actually reflect inequalities in health care since such health outcomes are affected by many other factors. Therefore, studies on socioeconomic differences in the quality of health care should also consider process indicators like patient-provider interaction.

#### Social relationships

Social relationships may influence adherence to medical treatment, help-seeking behavior, and *utilization* of health services [11]. In the Behavioral Model of Health Care Utilization developed by Ronald Andersen [31] social relationships (integration in social networks and social support) are conceptualized as individual predisposing factors that can predict use of health care services. This use can be seen as the final stage of a decision making process that shapes help-seeking behavior. According to Siegrist [32] this process starts with the recognition of a symptom/problem followed by the decision whether self-treatment might help. In the next stage advice is sought from the partner, close relatives, friends or other persons in the lay system (i.e. instrumental support, see above). Subsequently, a decision is made whether health care services are utilized or not. In this regard, social relationships play a significant role in the way health complaints are recognized and interpreted. Hence, analysis of social relationships can help to understand variations in health care utilization. Furthermore, social support can affect medical treatment outcomes like survival, recovery or quality of life.

#### Social capital

A promising avenue for health services research is to analyze the role of social capital in the delivery of health care. As outlined above, social capital can be conceptualized and measured on different levels [18]. On the meso-level social capital can be defined as features of organizations such as networks, norms and social trust that act as resources for individuals and facilitate coordination and cooperation of mutual benefit. Health care organizations (e.g. hospitals) with greater social capital can be characterized as having social relations between their members that are based on trust, mutual understanding, common convictions, and shared values. There is evidence for a relationship between social capital within hospitals and hospital performance as well as quality of care [33, 34]. However, further research is needed to evaluate the functionality of social capital in health care organizations in the delivery of high-quality coordinated care. Furthermore, on the individual level, patient’s trust in the health care system and services is an important factor that can have an impact on utilization of health care.

#### Work stress

Job stress is highly prevalent among health care providers and can not only affect their health but also their performance [35]. Studies indicate a higher risk of medical errors and suboptimal patient care among stressed physicians. Thus, working conditions among health care providers are an important determinant of quality. However, the majority of corresponding investigations are not based on a theoretical model of job stress [36]. Such models (like the ERI model and the demand-control model outlined above) help to identify stressful working conditions, to understand possible consequences, and to develop interventions to improve working conditions. A study among clinicians in surgery shows that working conditions of about a quarter of the doctors were characterized by an effort-reward imbalance. 22 % of them had job strain according to the demand-control model, i.e., they were confronted with high demands, yet had a low degree of control [37]. Moreover, significant associations between both job stress models and quality of care among the clinicians were found [36]. Based on these models interventions to improve psychosocial work environment in hospitals were developed and successfully implemented [38]. Thus, the availability and adequacy of occupational health services are important issues for health services research.

## Summary

The main argument of this paper was that concepts of social epidemiology can make a useful contribution to health services research because the underlying social factors do not only influence health but are also related to health care. Social inequality indicators like education or income have an impact on access to health care as well as on utilization and quality of health care in different countries/health care systems. Social relationships (integration in social networks and social support) influence adherence to medical treatment, help-seeking behavior, utilization of health services, and outcomes. Social capital in health care organizations (i.e. social relations that are based on trust, mutual understanding, common convictions, and shared values) is an important factor for the delivery of high-quality coordinated care. The psychosocial work environment of providers is a relevant determinant of quality of health care. Therefore, the theoretical considerations behind factors like social inequalities, social relationships, social capital and work stress can enrich health services research because theory helps to specify the research question, to clarify methodological issues, to understand how social factors are related to health care and, to develop and implement interventions [4].
